# Effect of low intensity photobiomodulation associated with norbixin-based poly (hydroxybutyrate) membrane on post-tenotomy tendon repair. In vivo study^1^

**DOI:** 10.1590/s0102-865020200030000003

**Published:** 2020-05-22

**Authors:** Lízia Daniela e Silva Nascimento, Renata Amadei Nicolau, Antônio Luiz Martins Maia, Kárita Francisca e Silva Nascimento, José Zilton Lima Verde Santos, Rayssilane Cardoso de Sousa, Luiz Fernando Meneses Carvalho, Vicente Galber Freitas Viana

**Affiliations:** I Fellow PhD student, Postgraduate Program in Biomedical Engineering, Universidade do Vale do Paraíba (UNIVAP), Sao Jose dos Campos-SP. Assistant Professor of Kinesiology, MSc, Health Sciences Center, Physical Therapy, Universidade Estadual do Piauí (UESPI), Teresina-PI, Brazil. Conception, design, intellectual and scientific content of the study; acquisition and interpretation of data; technical procedures; manuscript preparation and writing.; II PhD, Collaborator, Postgraduate Program in Biomedical Engineering of the Research and Development Institute, UNIVAP, Sao Jose dos Campos-SP, Brazil. Conception, design, intellectual and scientific content of the study; interpretation of data; critical revision; final approval.; III Associate Professor of Physiology, Health Sciences Center, and Researcher of the Biotechnology and Biodiversity Core, UESPI, Teresina-PI, Brazil. Technical procedures.; IV Speech and Hearing Therapist, Department of Social Assistance and Citizenship of the State of Piauí (SASC), Teresina-PI. Specialist in Language (UNIFOR), Fortaleza-CE, Brazil. Statistical analysis, critical revision.; UNIFOR, Fortaleza, CE, Brazil; V Assistant Professor, MSc, Center of Health Sciences, Medicine and Physical Therapy, UESPI. Professor, Centro Universitário Uninovafapi, Teresina-PI, Brazil. Histopathological examinations.; Centro Universitário Uninovafapi, Teresina, PI, Brazil; VI Fellow PhD student, Postgraduate Program in Biotechnology, Universidade Federal do Piauí (UFPI), Teresina-PI, Brazil. Membrane production.; VII PhD, Postgraduate Program in Material Engineering, Federal Institute of Education, Science and Technology of Piaui (IFPI), Teresina-PI, Brazil. Membrane production.; VIII Professor, Postgraduate Program in Material Engineering (PPGEM), IFPI, Teresina-PI, Brazil. Membrane production.

**Keywords:** Achilles Tendon, Membranes, Low-Level Light Therapy, Tenotomy, Wound Healing, Rats

## Abstract

**Purpose:**

To evaluate the in vivo response of photobiomodulation therapy associated with norbixin-based poly(hydroxybutyrate) membrane (PHB) in tenotomized calcaneal tendon.

**Methods:**

Thirty rats were randomly allocated to six groups (n=5 each): LED groups (L1, L2 and L3) and membrane + LED groups (ML1, ML2 and ML3). The right calcaneal tendons of all animals were sectioned transversely and were irradiated with LED daily, one hour after surgery every 24 hours, until the day of euthanasia. At the end of the experiments the tendons were removed for histological analysis.

**Results:**

The histological analysis showed a significant reduction in inflammatory cells in the ML1, ML2 and ML3 groups (p=0.0056, p=0.0018 and p<0.0001, respectively) compared to those in the LED group. There was greater proliferation of fibroblasts in the ML1 (p<0.0001) and L3 (p<0.0001) groups. A higher concentration of type I collagen was also observed in the ML1 group (p=0.0043) replacing type III collagen.

**Conclusion:**

Photobiomodulation in association with norbixin-based PHB membrane led to control of the inflammatory process. However, it did not favor fibroblast proliferation and did not optimize type I collagen formation in the expected stage of the repair process.

## Introduction

Calcaneal tendon rupture is the most frequent tendon lesion in high physical activities of intensity sports such as running and jumping, affecting not only athletes, but also sedentary, and may limit the function and ability of patients to perform their activities of daily living, since it plays an important role in walking^1^ ^–^ ^5^. It affects young and middle-aged individuals more frequently, but recent studies have indicated its increasing incidence among active elderly^4^ ^,^ ^6^. Treatment options for this lesion include conservative and surgical (open or percutaneous) measures; however, these options are still controversial and debated in the scientific community regarding the recurrence of rupture and other complications^4^ ^,^ ^7^ ^,^ ^8^.

After the lesion, the process of repairing the tendinous tissue occurs in three distinct phases: tissue inflammation, cell proliferation and extracellular matrix remodeling. The inflammation happens to protect the organism, eliminate and dilute harmful agents at the affected area and promotes increase of the capillary permeability and vasodilation, leading to the formation of edema. In the proliferation phase, there is an increase in the number of components of the extracellular matrix and collagen type III. In the remodeling phase, type III collagen fibers decrease and type I collagen fibers increase and there is longitudinal realignment^9^ ^,^ ^10^.

Among the therapeutic resources used in the repair of tendinous tissue after rupture, low intensity photobiomodulation in previous studies has demonstrated benefits such as improved distensibility and increased resistance to ruptures due to increased ATP synthesis, cell proliferation, vascularization and proliferation of fibroblasts, as well as collagen synthesis^11^ ^–^ ^13^. Therapeutic LEDs promote biomodulation of biological tissue, characterized by emitting visible light or in the near infrared region, not coherent and non-collimated^14^ ^,^ ^15^.

In addition to biomodulatory resources such as LED therapy, natural biological membranes or those synthesized by tissue engineering have been used in the repair of the calcaneal tendon to improve healing after rupture^16^ ^–^ ^18^. These biomaterials have biocompatible characteristics and are biodegradable, allowing total or partial regeneration of biological tissue. Within this context, a norbixin-based poly(hydroxybutyrate) (PHB) membrane was produced for use in the repair of various tissues, including tendons^19^. The PHB is a bioactive and biomimetic polymer that may originate from natural sources or be synthesized by bacteria from compounds such as butyric acid^20^ ^–^ ^22^. Norbixin, a water-soluble substance, is one of the compounds extracted from the urucum seed (Bixa orellana L.), an evergreen plant native to tropical South America. This component exhibit several useful bioactivities, reinforcing its importance as a medicinal and pharmaceutical asset, in addition to its common use as a food colorant^23^ ^,^ ^24^. In the literature there are several studies citing the efficacy of norbixin as an antioxidant, protecting cells against free radicals, corroborating Santos *et al*.^25^ statement about the absence of genotoxic, teratogenic, mutagenic or clastogenic effects of this substance in animals.

Thus, this study was conducted taking into consideration the satisfactory results of photobiomodulation in experimental studies in recent years and the interest in understanding the interaction between LED irradiation and the norbixin-based PBH membrane in the process of tendon repair.

## Methods

### Experimental groups

The present study was approved by the Animal Research Ethics Committee (CEUA), Universidade Estadual do Piauí (UESPI) under protocol number 14776/16. Thirty male wistar rats (*Rattus norvegicus*), approximately 10 weeks old, clinically healthy, weighing between 250 and 300g and kept individually in polypropylene cages daily cleaned, were used. The animals were fed with feed and water *ad libitum* under dark light cycle of 12h/12h, with a controlled temperature of 24°C. The animals were randomly separated into six groups (n=5 each) and euthanized in three distinct time periods (7, 14 and 21 days): L1 (LED therapy, euthanasia in 7 days), L2 (LED therapy, euthanasia in 14 days), L3 (LED therapy, euthanasia in 21 days), ML1 (membrane + LED therapy, euthanasia in 7 days), ML2 (membrane + LED therapy, euthanasia in 14 days) and ML3 (membrane + LED therapy, euthanasia in 21 days).

### Surgical procedure

The entire surgical procedure was performed as described by Nascimento *et al*.^19^. Initially the rats received subcutaneous injection of 0.04 mL/g of atropine for sedation and then were anesthetized intramuscularly using 10% ketamine and 2% xylazine at a dose of 0.1 mL for each 100g of body weight. The right calcaneal tendon was exposed through a 1 cm incision in the skin and total tenotomy was performed in all animals. Among the 15 animals belonging to the LED groups (L1, L2 and L3), there was no fixation of the tendon stumps. In the animals belonging to the Membrane +LED (n=15) groups (ML1, ML2 and ML3), a fragment of approximately 6 × 3 mm length and width, respectively, of the PHB membrane with norbixin previously produced in the Materials Engineering Master's course of the Federal Institute of Piauí (IFPI) was glued with methacrylate to the two portions of the sectioned tendon, joining them. Subsequently, in all animals, a cutaneous suture was performed with 4-0 polyester monofilament yarn. After the surgical procedure, the animals were kept in cages (n=5) according to the experimental groups, receiving analgesia by gavage. No surgical site infection was observed and the area of the skin where the incision was made showed no dehiscence.

### Experimental protocol

The LED groups (L1, L2 and L3) and membrane + LED (ML1, ML2 and ML3) groups were submitted to treatment with photobiomodulation initiated one hour after the procedure and repeated every 24 hours (once a day). For this purpose, the animals were placed on a table in ventral decubitus position and manually immobilized. The right hind legs received transcutaneous irradiation in a timely manner in direct contact with the tenotomized region, forming an angle of 90° in relation to the lesion area. The equipment used in the study was a LED prototype (660 ± 20nm), produced at the Research and Development Institute (IP&D), UNIVAP, with the following parameters: continuous emission, power of 14 mW, time of 214.3s (∼4 minutes), coupled collimator area of 0.5 cm2 in diameter, obtaining an energy density of 6 J/cm^2^ at the single point of application, irradiance of 0.028W/cm^2^ and energy of 3J. The cumulative energy doses and creep were 18 J and 36 J/cm^2^ (groups L1 and ML1), 39 J and 78 J/cm^2^ (groups L2 and ML2) and 60 J and 120 J/cm^2^ (groups L3 and ML3), respectively (Table 1). Prior to the start of the experiments, the LED equipment was verified using a power meter (Model 13 PEM 001/J, MellersGriot, Netherlands) at a dosage of 6 J/cm². The pen of the device was protected with plastic film before each irradiation. The animals of the membrane group were also manipulated during the same time, but the equipment was turned off.

**Table 1:** Photobiomodulation parameters.

Parameters	Values
Equipment	Prototype LED - IP&D (UNIVAP)
Wavelength (nm)	660 ± 20
Power (mW)	14
Power Density (W/cm^2^)	0.028
Energy (J)	3
Energy Density (J/cm^2^)	6
Beam Area (cm^2^)	0.5
Irradiation time per point (s)	214.3s
Number of points	1
Mode of application	Direct Contact
Number and frequency of sessions	6 sessions (groups L1, ML1)*
	13 sessions (groups L2, ML2)*
	20 sessions (groups L3, ML3)*
	*to every 24 hours, starting one hour after the surgical procedure.
Cumulative energy dose (J) and total energy density (J/cm^2^)	18 and 36 (groups L1 and ML1)
	39 and 78 (groups L2 and ML2)
	60 and 120 (groups L3 and ML3)

### Euthanasia

The animals were euthanized individually on the seventh day (groups L1 and ML1), the fourteenth day (groups L2 and ML2) and the twenty-first postoperative day (groups L3 and ML3) using excessive dose of sodium thiopental anesthetic. Then, the treated tendons were removed and fixed in a 10% formaldehyde solution for histological processing.

### Histological technique

After fixation for 24 hours, the tendons were dehydrated in a series of 70%, 80%, 90% and absolute alcohol solution and included in paraffin; then, the blocks were made to be cut into semi-series microtome with sections 5 µm thick and stained with H&E (Hematoxylin and Eosin) (n=2) and Picrossírius Red (n=2).

**Figure 1:**
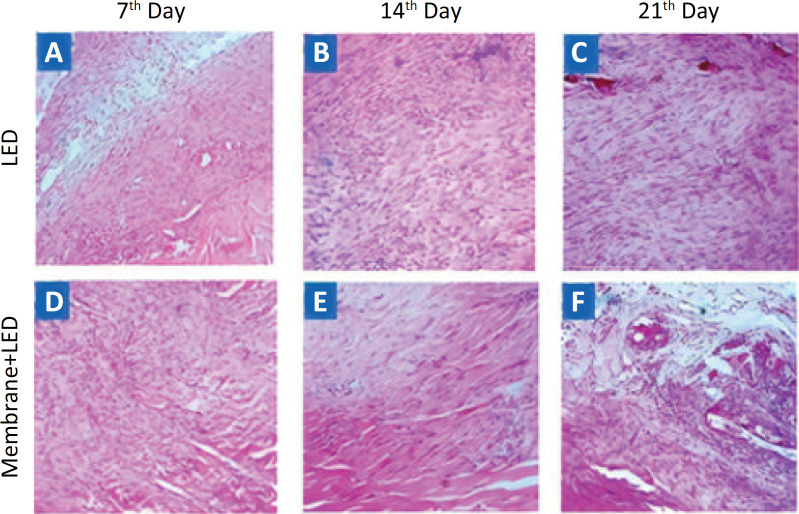
Representative images of inflammatory cells and fibroblasts in the calcaneus tendon in the LED **(A-C)** and Membrane + LED **(D-F)** groups during the experimental periods of 7, 14 and 21 postoperative days, stained with HE (x100).

The count of inflammatory cells and fibroblasts on the slides stained with HE (Hematoxylin & Eosin) was performed with the Zeiss microscope coupled to a Zeiss camera using a x20 objective with a x0.5 increase and 5mb resolution. Subsequently, Image J software was used in five fields per slide at x200 magnification and manual counting of the cellular nuclei was performed, which is the average score obtained in the fields determined by the single score for each sample from each experimental group (Fig. 1).

**Figure 2:**
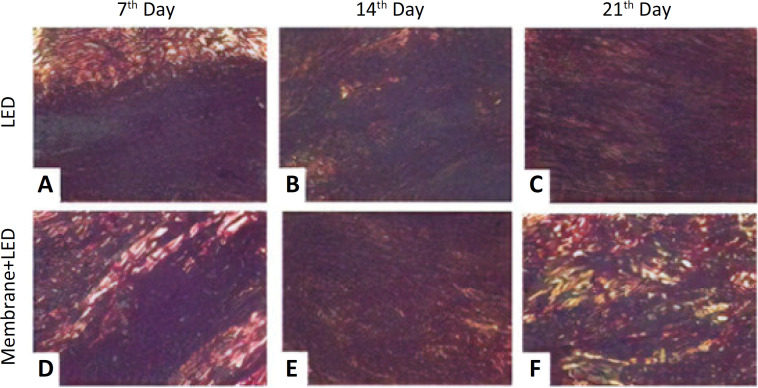
Histological aspects of collagen fibers in the LED **(A-C)** and Membrane+LED **(D-F)** groups on the 7, 14 and 21 postoperative days, stained with *picrosirius red* (x100).

The slides stained with Picrossirius Red were analyzed using a Leica® DM 2000 polarized light microscope and DFC 295 camera microphotography. Image J Pro-plus software was used to determine the percentage of type I (red) and type III (green) collagen fibers in the area corresponding to the calcaneus tendon repair (Fig. 2).

### Statistical analysis

The statistical analysis was performed with the GraphPad Prism program, version 5.0 (GraphPad, California, USA), using the Kolmogorov-Smirnov test. The Man-Whitney test was used for group comparisons, while the Kruskal-Wallis test and Dunn's post-test were used for intragroup analysis. All levels of significance were set at 5% (p <0.05).

## Results

### Quantitative analysis of inflammatory cells and fibroblasts

In the intragroup analysis of group L, a significant difference was found during the periods of 7-14 days (p=0.0021) and 7-21 (p=0.0021). In the ML group, a significant decrease of inflammatory cells was observed only in the period of 7-14 days (p=0.0018) (Table 2).

**Table 2:** Intra-group evaluation of the mean inflammatory cell count (mean ± standard error).

Groups	Period (days)			IE		
	7	14	21	p (7-14)	p (7-21)	p (14-21)
LED	*99,63 ± 2,61*	*90,90 ± 3,48*	*87,00 ± 2,09*	* *0,0021*	* *0,0021*	*ns*
Membrane+LED	*86,85 ± 2,88*	*78,15 ± 1,50*	*72,85 ± 2,07*	*ns*	* *0,0018*	*ns*

IE= Intra-group evaluation

* = Significant difference;

ns= Non-significant difference.

**Figure 3:**
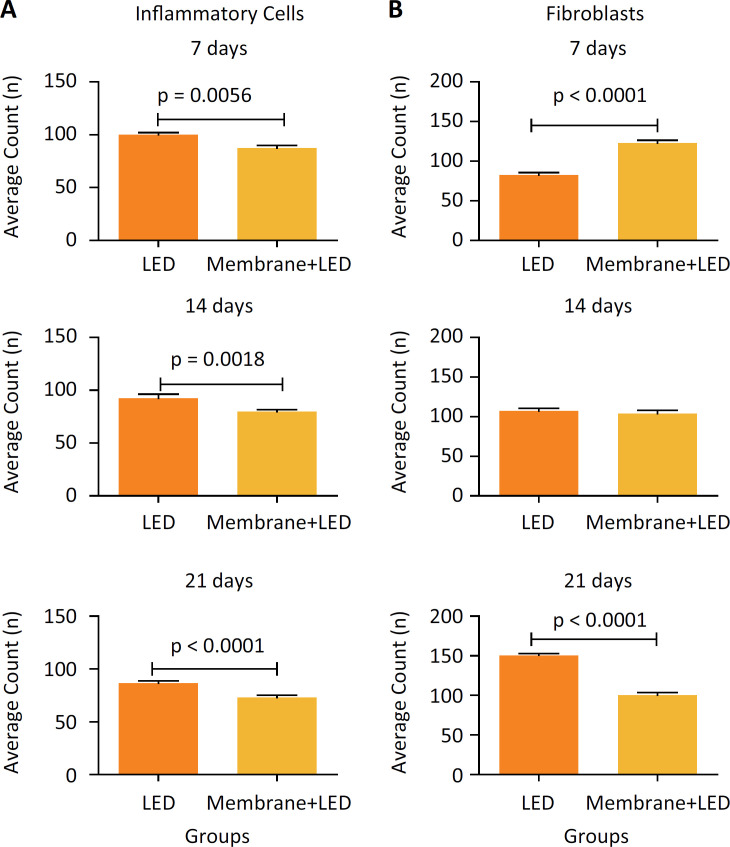
Analysis between the groups of the mean inflammatory cells count of and mean fibroblasts count.

The analysis between the groups showed that during all time periods, the ML group showed a significant reduction on the 7 (p=0.0056) and 14 (p=0.0018) days and an extremely significant reduction in 21 days (p<0.0001), when compared to the isolated L group (Fig. 3A).

The count of the mean number of fibroblasts in an intragroup analysis showed that group L exhibited an extremely significant increase during all experimental periods (p<0.0001). While in the ML group, there was a significant reduction during the periods of 7-14 (p=0.0010) and 7-21 days (p=0.0010) (Table 3).

**Table 3:** Intra-group evaluation of the fibroblast count (mean and ± standard error).

Groups	Period (days)			IE		
	7	14	21	p (7-14)	p (7-21)	p (14-21)
LED	*82.00 ± 2.75*	*105.3 ± 3.11*	*150.6 ± 2.36*	* *<0.0001*	* *<0.0001*	* *<0.0001*
Membrane+LED	*121.6 ± 4.03*	*101.9 ± 3.77*	*99.72 ± 3.74*	* *0.0010*	* *0.0010*	*ns*

IE= Intra-group evaluation;

* = Significant difference;

ns= Non-significant difference.

In the analysis between the groups it was possible to observe that in 7 days the ML group displayed an extremely significant increase (p<0.0001) when compared to the L group (Fig. 3B). In 14 days, no significant difference was found. During the last experimental period (21 days), it was found that group L showed a high mean fibroblasts count, with an extremely significant difference (p<0.0001) in relation to group ML.

### Quantitative analysis of collagen concentration

The intragroup analysis of the L group showed a significant increase in type I fibers during the periods of 7-21 (p=0.0020) and 14-21 (p=0.0020) and a significant decrease in type III fibers during the periods of 7-14 and 7-21 days with p=0.0020. In the ML group, there was no significant difference in the three time periods (Table 4).

**Table 4:** Intra-group evaluation (mean and standard error).

Groups / TC (%)		Period (days)			IE		
		7	14	21	p (7-14)	p (7-21)	p (14-21)
LED	CTI	*9.47 ± 2.68*	*39.22 ± 7.03*	*47.11 ± 8.42*	*ns*	* *0.0020*	* *0.0020*
	CTIII	*90.53 ± 2.68*	*60.78 ± 7.03*	*52.89 ± 8.42*	* *0.0020*	* *0.0020*	*ns*
Membrane+LED	CTI	*37.99 ± 8.45*	*49.55 ± 10.20*	*24.98 ± 9.05*	*ns*	*ns*	*ns*
	CTIII	*62.01 ± 8.45*	*50.45 ± 10.20*	*75.03 ± 9.05*	*ns*	*ns*	*ns*

CT= Collagen types;

CTI= Collagen type I;

CTIII= Collagen type III;

IE= Intergroup Evaluation;

* = Significant difference;

ns= Non-significant difference.

**Figure 4:**
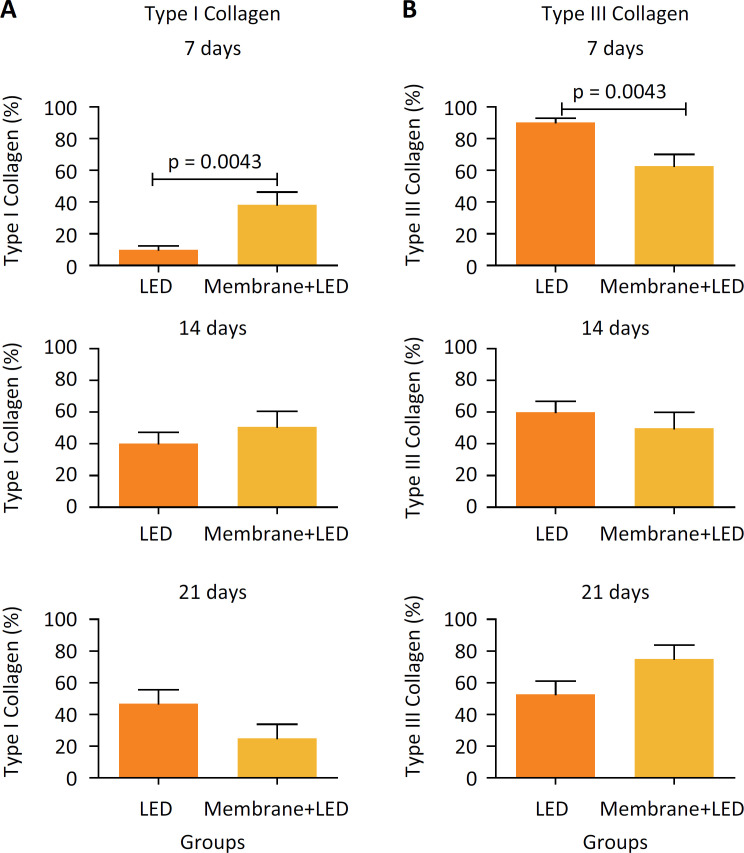
Intergroup analysis of the percentage of type I and type III collagens (mean ± standard error).

Figure 4 shows the intergroup analysis of the variation (%) of type I collagen fibers (Fig. 4A) and III collagen fibers (Fig. 4B) during the periods of 7, 14 and 21 days. In the analysis of type I collagen, in 7 days it was possible to verify that group L showed a significant reduction (p = 0.0043) in relation to group ML. In 7 days, the lowest mean of group L was verified when compared with ML, while in 21 days, group L demonstrated the highest mean of type I collagen. However, there was no significant difference in the percentage of type I collagen during these periods. The evaluation of type III collagen fibers showed a significant decrease in group ML on day7 (p-value = 0.0043) compared to group L. During the other experimental periods, no significant differences of type III collagen between the analyzed groups were observed.

## Discussion

The increased incidence of chronic lesions of the calcaneus tendon observed in recent decades has been motivating studies that address therapeutic alternatives to improve and/or accelerate the repair of this condition^16^ ^,^ ^26^, since this lesion may result in severe functional impairment and a drastic reduction in quality of life^27^. In this context, studies that employed biocompatible membranes or photobiomodulation obtained promising results^13^ ^,^ ^14^ ^,^ ^16^ ^,^ ^17^. However, so far there has been no association between these techniques. Thus, this study aimed to associate the norbixin-based PHB membrane with LED therapy in the process of tendon repair.

The inflammatory process lasts about 7 days, with the formation of hematoma in the region of the lesion during the acute phase. Inflammatory cells (neutrophils, macrophages and platelets) then begin to release cytokines and growth factors that will stimulate the proliferation of cells and substances in the next step^9^ ^,^ ^28^. In this study, it was observed that the acute inflammatory process in the LED group occurred more pronouncedly than in the ML group; however, there was a gradual decrease in the amount of inflammatory cells in both groups. This leads us to think that the anti-inflammatory characteristic of the membrane in the ML group was responsible for the result found, since all the experimental groups received the same treatment with of LED. It has been observed that animals treated with LED after tendinous lesions show improvement in inflammatory conditions when compared to non-irradiated animals^29^ ^,^ ^30^. Such effect on inflammation, obtained by LED therapy, is related to increased peripheral microcirculation, modification of membrane potential, resulting in anti-inflammatory effects^29^ ^,^ ^31^. It is known that norbixin, a component of the membrane, has anti-inflammatory properties, as already demonstrated by Viana *et al*.^32^ in an *in vitro* induced peritonitis model, thus justifying the reduced acute inflammation in the Membrane + LED groups.

Unlike what happened with inflammatory cells, the number of fibroblasts gradually increased in the LED group, while it decreased in the Membrane + LED group over the experimental period. It is known that the proliferative phase of the tendon repair is characterized by increased proliferation of fibroblasts^33^ ^,^ ^34^, which are responsible for collagen synthesis. Both the increase in the number of fibroblasts and the synthesis of collagen are closely related to the process of tissue repair, especially after-tenotomy. The result found in the ML group demonstrated that the presence of the membrane may have partially inhibited the proliferative phase. One cannot say that the implanted biomaterial constituted a barrier for the penetration of light emitted by LED, because the membrane was located on the deepest surface of the tendon, that is, the light did not need to pass through it before reaching the tendon.

According to Park *et al*.^35^ and Misir *et al*.^36^, reducing the inflammatory process in the early stages of repair in tendinous lesions may result in a better quality repair. On the other hand, the greater amount of fibroblasts can optimize the repair process by synthesizing collagen in the extracellular matrix of the tendinous tissue^37^. The types of collagens produced during the tissue repair process are initially type III (immature), whose fibers are not yet parallel-oriented, and consists of smaller and weaker fibers with low strength and then type I (mature), whose fibers are more resistant and longitudinally oriented^9^ ^,^ ^28^ ^,^ ^38^.

Regarding collagen, a gradual replacement of type III collagen by type I collagen was found in group L throughout the experimental period. In the ML group, the results showed that in the first two weeks the amount of type I collagen increased and type III decreased, while in the third week there was a decrease in type I collagen and an increase in type III collagen. The tendinous tissue produced after healing with abundance of type III collagen is much less organized, resulting in loss of structure and decreased mechanical resistance and it is believed that the increase in the production of type III collagen, compared to type I, leads to the formation of adhesion sites, characterized by inadequate lubrication between the tendon and surrounding tissues, causing friction and pain, limiting the tendon sliding and reducing mobility. In addition, the higher concentration of collagen type III increases the risk of reoccurrence of rupture, since it is related to reduced mechanical strength^34^ ^,^ ^39^. According to Muller *et al*.^10^, for the optimization and rapid healing of tendons, a high expression of type I collagen is extremely important, which did not occur in group ML of this study.

Notably, group L exhibited a higher amount of collagen than group ML; from this, it is possible to infer that the association between the norbixin-based PHB membrane and LED therapy did not produce a quality tendon repair. This non-optimization of therapeutic effects in the associated group (LM) may be due to the oxidative degradation of norbixin after the interaction with electromagnetic irradiation promoted by LED therapy. In agreement with these findings, Alves *et al*.^40^ used a membrane composed of norbixin associated with photobiomodulation (λ 780 nm) in bone repair and found that the association of this modality failed to improve the bioactive properties of the membrane and vice-versa.

## Conclusions

The use of norbixin-based PHB membrane associated with low-intensity LED therapy in the treatment of total calcaneal tenotomy of rats reduced the levels of inflammatory infiltrate in the lesion. However, it did not favor the proliferation of fibroblasts in the first two weeks and did not optimize the formation of type I collagen in the expected stage of the repair process. The present study indicates that there was little effectiveness in the simultaneous use of the two therapeutic elements when compared to the isolated use of LED therapy.
